# 3D Kinematics of Male and Female Soccer Players for a Variety of Game-Specific Skills

**DOI:** 10.1155/2024/9588416

**Published:** 2024-01-08

**Authors:** Danyon Loud, Paul Grimshaw, Richard Kelso

**Affiliations:** ^1^School of Electrical and Mechanical Engineering, The University of Adelaide, Adelaide, SA, Australia; ^2^College of Health and Life Sciences, Hamad Bin Khalifa University, Doha, Qatar

## Abstract

Soccer is played by a variety of individuals with varying abilities. The complicated lower limb movements involved within the game often lead to knee and ankle injuries, with anterior cruciate ligament injuries being the most severe with regard to rehabilitation time and ongoing health risks. This research explores the biomechanical kinematics of male and female soccer players on synthetic grass to determine whether trends in lower limb biomechanics over a variety of movements could explain injury risk. Both male and female players (*n* = 10) aged between 19 and 24 years performed running-based and stationary-start movements. Biomechanical measurements at the hip, knee, and ankle were recorded. Observations showed that specific differences in joint angles were largely dependent on the movements performed; however, for male players, on average, across all movements, 84.6% and 72.6% of the variation in joint angles could be explained by internal/external rotation at the hip and knee, respectively. For female players, internal/external knee rotation, as well as hip abduction and adduction, accounted for 83.6% and 80.2% of the variation in joint angles, respectively, across all the tested movements. This highlights the importance of hip mechanics and knee alignment for players when performing a variety of movements.

## 1. Introduction

**Figure 1:**
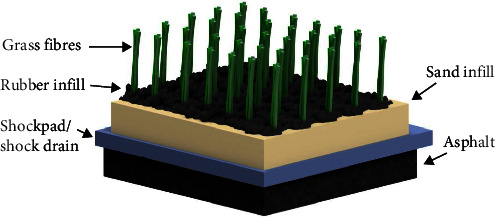
Fourth-generation artificial grass (image generated in Autodesk Inventor).

With over 265 million players worldwide, soccer has a large cultural and economic impact [1]. While traditionally played on natural grass, the economic benefits of artificial grass have seen an adoption of these playing surfaces around the world. Lower maintenance costs and increased pitch availability means artificial grass surfaces can save clubs approximately $2,255 AUD annually ($3074 AUD in 2023 dollars based on the consumer price index) as well as 515 man-hours in maintenance and upkeep [2]. As shown in Figure 1, fourth-generation artificial grass consists of tufted fibre, a rubber performance infill, a supporting sand infill, and a shock absorption layer. Previous generations of artificial grass lacked these infill layers, with players 2.5–4.5 times as likely to incur injuries on first- and second-generation artificial turf when compared to natural grass [3, 4]. While later generations of artificial grass provide no direct injury risk to players, they do have preconceived behaviours when playing on artificial grass, which could impact their lower limb biomechanics [5]. Potthast [6] found that along with changes in a player's behaviour due to changes in the surface type, there were also biomechanical differences, particularly for ankle eversion when they performed an instep kick on natural and artificial grass. This change in player biomechanics could have implications for the athlete, placing them at risk of lower limb injury.

The knee and ankle are the two primary injury locations for soccer players, accounting for approximately 40% of injuries [7]. A tear of the anterior cruciate ligament (ACL) in the knee has numerous negative impacts on an individual, including significant economic costs, a large rehabilitation period, and an increased risk of Osteoarthritis later in life [8]. This high injury risk coincides with the reported 70%–80% of ACL injuries occurring in non-contact situations [9]. In a systematic video analysis of 39 non-contact ACL injuries of professional players, Waldén et al. [10] found that pressing actions (often involving a sidestep cut to tackle an opponent), followed by regaining balance after kicking or landing after heading the ball, were the most common causes for ACL injury.

A 16-year analysis of collegiate level soccer players noted that female soccer players experienced 0.28 ACL injuries per 1,000 hr (training and games) compared to 0.09 ACL injuries per 1,000 hr in men's soccer [11]. The cause of this disparity among sexes is disputed among the literature. Studies have shown that the intercondylar notch in the knee is smaller in females than in males, whereas others highlight the influence of the menstrual cycle or the difference in biomechanics, notably the development of knee valgus, between sexes [12, 13]. While there are significant differences in male and female injury rates, there exists limited literature that examines potential causes of these differences, particularly the biomechanics of male and female soccer players that occur over a broad range of movements in a game-specific environment with minimal outside interference.

Previous literature has focussed on single movements of soccer players, such as changes of direction [14–17], kicking [18–21], and jumping [22–24]. These studies utilised motion capture software to analyse the position and kinematics of the players' lower limbs. In these studies, the motion capture systems were set up in an indoor environment, with Thomas et al. [15] opting to use an indoor, artificial grass patch as opposed to a traditional laboratory floor utilised by other researchers. With regard to testing methods, there are also significant differences between studies with Landry et al. [14], Condello et al. [16], Thomas et al. [15], and Pollard et al. [17] using 3D kinetic and electromyographic analysis, force plate analysis and a motion capture analysis respectively to deliver varying results. While some studies found kinematic differences between sexes at the hip [14] and others at the knee [15], some studies found no differences between male and female players [17], highlighting the influence of testing methods. Similar trends were noted in the analysis of kicking kinematics, with some studies noting significant differences at the hip joint [20], a factor that aligns with the findings of Landry et al. [14] for a change of direction analysis, and other studies finding no significant differences in the position of the standing leg between sexes [21]. Interestingly, a majority of the literature examines differences during an instep kick, a kicking technique often used for shooting or longer passes; however, Althoff and Hennig [25] suggest that female players are beginning to rely on shorter passes, utilising the side-step kicking motion rather than the instep kicking motion. While present literature examines the biomechanical differences in a reliable and repeatable manner, ACL injuries are not limited to one movement, as evidenced by Waldén et al. [10], and players appear to behave differently when playing on different surfaces [5].

Therefore, the aim of the present study is, through the application of 3D motion capture, to characterise the biomechanics of male and female soccer players over a variety of movements that are performed on a game-specific artificial grass surface. Due to the link between knee valgus and the potential for ACL injury risk, particularly for female players, the hypothesis for this research was that knee valgus would be the most influential biomechanical characteristic among female soccer players.

## 2. Materials and Methods

### 2.1. Participants

Ten amateur soccer players (five male and five female) were recruited for this study to perform eight distinct movements. The participants were grouped by their pre-acknowledged sex as either “male” or “female.” Each player was required to be within the age range of 18–25 years, have a minimum of 5 years of playing experience at a club level, as well as no previous injuries to their ACL in either leg. The five male soccer players (1.80 ± 0.06 m height, 76 ± 8 kg mass, 23 ± 2 years of age, 12 ± 3 years playing experience) and the five female soccer players (1.62 ± 0.11 m height, 62 ± 10 kg mass, 21 ± 2 years of age, 12 ± 5 years playing experience) provided their informed consent to participate in this study that was approved by The University of Adelaide Human Research Ethics Committee.

### 2.2. Tasks

Prior to testing, the players were screened with a questionnaire detailing injury history, and they were given an overview of tasks and the surface type. Based on this information, players were instructed to wear their choice of soccer boots to suit the playing surfaces. By instructing players to wear their own footwear, any unfamiliarity with the boot feel was avoided. Players completed their own 15-min warm-up prior to performing each of the following actions four times:

- Straight-line run;
- Run and stop;
- Run and single leg 180° turn;
- 45° plant-and-cut;
- Two legs vertical jump;
- A vertical jump, followed by a 45° take-off (jump exit);
- Kicking with the instep and the side foot.

Straight-line running, sudden stops, and changes of direction were all categorised in defensive “pressing,” while landing from jumps and kicks were all labelled as potential ACL injury mechanisms [10].

Prior to each test, players were told what movement they were to be performing and, where applicable, informed of the area within the capture frame where the movement must take place. Players were given a visual demonstration of a “successful” trial for each movement; however, no further instruction was given to specific technical aspects of each movement to ensure the results obtained were as true to the players' normal actions as much as possible. The players' approach speed for applicable actions such as running and change of direction movements was recorded by observing their average horizontal speed of the left anterior superior iliac and right anterior superior iliac markers in the motion capture software. If a participant's speed was particularly low (approximately 1 m/s) compared to previous trials, they were questioned for possible fatigue and encouraged to take a break. Trials that were performed at speeds outside of this lower threshold were included in the study. No pre-determined rest periods were prescribed between each movement; however, participants were questioned for mental and physical wellbeing before each action commenced. Motion capture results were checked after each test, and in trials where a particular marker was not obtained throughout the entire movement, the trial was repeated (up to a maximum of six total trials for each movement). As the participants were considered amateur players, it was assumed there would be significant kinematic differences between their dominant and non-dominant foot, particularly for kicking tasks, thus, participants were instructed to perform actions with their dominant foot, even change of direction movements.

### 2.3. Data Collection

**Figure 2:**
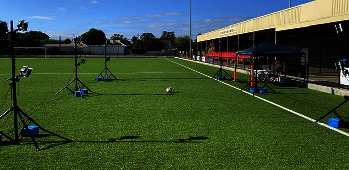
The 12-camera motion capture system on the artificial grass playing surface.

Kinematic data were recorded using a 12-camera VICON motion capture system (VICON, Oxford, UK) operating at 100 Hz, as recommended by the motion capture company LOGEMAS (LOGEMAS, Queensland, Australia) for outdoor motion capture. Sixteen 14 mm reflective markers were attached to each participant's lower limbs as per the VICON Plug-In-Gait marker set with marker clusters replacing individual markers on the shank and thigh segments. Players wore skin-tight clothing, and double-sided tape was used to adhere the markers to bony landmarks. Rigid sports tape was used to secure the marker clusters to the participant's outer thigh and shin segments. Rigid marker tape was also used to secure a marker to the distal end of the participant's first phalange. This location was chosen so as not to impede with the instep or sidestep kicking motion. The remaining markers on the foot were attached to the medial and lateral malleoli and the calcaneus using double-sided tape and rigid tape if necessary. The TekScan F-Scan in-sole pressure system (TEKSCAN, Massachusetts, USA) was used to calculate the vertical ground reaction force of the players for each movement. This force data was used to correlate the initial point of ground contact and was not used to perform a kinetic analysis. The artificial grass testing surface, shown in Figure 2, consisted of a Max S yarn, a styrene-ethylene-butylene-styrene performance infill, a sand-supporting infill, and finally, a ShockPad draining and shock absorption system. The motion capture volume was approximately 8 m × 4 m × 2 m, with players having ample approach room.

Ankle inversion and eversion angles, as well as the plantar and dorsiflexion, were recorded. The internal/external rotation, varus and valgus rotation, and the flexion and extension at the knee were also calculated. Finally, the internal and external rotation and abduction and adduction of the hip were collected. The linear velocity, in the direction of motion, of the player's hip centre was recorded for each running-based trial.

While movements at the ankle have minimal direct impact on ACL strain, they have a significant impact on the stability of the lower limb, with Baez et al. [26] finding a correlation between an individual's ankle joint function and the biomechanics at the knee. The biomechanical values gathered at the knee can place the ACL under direct strain (knee valgus and internal rotation) or indirect strain by placing the athlete in the “position of no return,” as described by Ireland [27]. This “position of no return,” whereby the femur becomes internally rotated, and the tibia externally rotated, places the knee in extreme levels of valgus rotation and can place additional strain on the ACL. Hip adduction and internal rotation also lead to athletes adopting the “position of no return” [27]. Key joint angles, such as knee rotation, knee valgus, and hip rotation, were identified as key components with respect to ACL injury, and hence, they needed to be recorded and processed.

Each value was recorded at ground contact, where the vertical acceleration of the foot was at the minimum value. Each instantaneous point was utilised in the statistical analysis, while the average values for each gender are displayed in Tables S1–S6. This singular value was chosen as it resembled the foot-fixation often attributed to non-contact lower limb injuries. This point corresponded with the initial vertical ground reaction force calculated using the in-sole pressure sensors. The foot-fixation, coupled with the initial pressure, resulted in this instance becoming the most likely for a player to sustain an ACL injury. Joint angles recorded at this moment could be either positive or negative relative to the neutral position. Prior to data collection, participants were asked to perform a static T-pose in the centre capture frame. This was used as a calibration for the motion capture system as well as the basis for a neutral position for each subject.

### 2.4. Data Processing

Data were processed in VICON Nexus using a low-pass Butterworth filter with a 10 Hz cut-off frequency as used by similar studies [28, 29] and then analysed using VICON ProCalc to determine joint angles and 3D kinematics. Kinematic values at the ankle, hip, and knee were calculated using definitions provided by VICON, based on the analysis of Kadaba et al. [30] and Davis et al. [31].

### 2.5. Statistical Analysis

**Figure 3:**
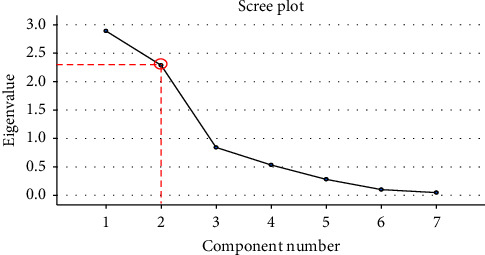
The scree plot from the PCA for the male sidekick movement with the inflection point denoted by the red circle.

The testing method resulted in the recording of eleven different data points across two sexes for eight different movements. To reduce the number of variables within the data set, a principal component analysis (PCA) was utilised. PCA is used to reduce the dimensionality of the data while retaining the variation within the data set [32]. By analysing the amount of variance in the data set that is attributed by each variable, the results can be explained in terms of key variables or principal components (PCs) rather than the entire variable list. A PCA was performed for each movement for both the male and female data to observe if there were variables that consistently provided the most impact on variance for each sex and movement. The PCA was performed in SPSS Statistics (IBM, New York, USA), and the data set was evaluated using the Kaiser–Meyer–Olksin (KMO) measure of sampling adequacy and Bartlett's test of sphericity. A KMO value close to 1 indicated that a PC would approximate the variance in the data, while a KMO of less than 0.5 indicated that the data reduction would not yield an accurate representation of results. Movements that resulted in a KMO of less than 0.5 were not reduced using the PCA; however, movements that yielded a KMO greater than 0.5 were rotated using a direct Oblimin rotation due to the nature of the variables, particularly the likelihood of some degree of correlation between lower limb biomechanics as suggested by Costello and Osborne [33]. A similar approach was adopted by Landry et al. [14], who selected an orthogonal rotation for their PCA. The subject-to-item ratio, described by Costello and Osborne [33] as the number of trials per variable tested, is a metric for observing the impact of sample size. In literature, it is common for subject-to-item ratios to be 10 : 1 or less. In this particular analysis, the ratio was 3.3 : 1, which lied between the 2 : 1 and 5 : 1 range used by 25.8% of studies [33]. The scree plot, shown in Figure 3, details a variable, in this case, a joint angle, as a component, as well as the associated eigenvalue for each component. This eigenvalue is derived from the transformation of each variable along the principal component, with a higher eigenvalue denoting a higher influence on the variance of data. The number of components analysed for each test was selected based on visual inspection as well as the eigenvalue for each variable. An inspection for each scree plot was undertaken for any key inflection points, as seen in Figure 3. It was at this inflection point where the number of components was selected. In instances where a clear inflection point could not be identified, an eigenvalue approach was utilised by selecting the number of variables with eigenvalues greater than 1, as suggested by Ringnér [32].

Due to the location of the inflexion point in Figure 3, two PCs were chosen for this particular movement. Once the number of PCs has been selected, a component matrix is constructed, as shown in Table 1. This matrix details the PCs and the corresponding impact of the individual biomechanical value, with a value closer to 1 signifying a higher influence on the variance. From Table 1, it can be deduced that, for Component 1, the internal/external rotation of the knee had the largest influence, while for Component 2, it was the knee varus/valgus for this movement. This methodology was used to deduce what biomechanical value had the most influence over a broad range of movements for both male and female players.

## 3. Results

Tables S1–S6 in the supplementary material detail key metrics and joint angles at the ground contact point for both male and female players over a variety of actions. Angles are measures relative to a stationary T-pose for each athlete and are listed as their mean value over the four trials, as well as a standard deviation. Tables S1 and S2 detail joint angles of the ankle joint for both males and females, respectively. A positive value for ankle rotation about the frontal plane axis denotes ankle inversion, while a negative value represents eversion. Similarly, positive angles represent plantarflexion, while negative angles represent dorsiflexion. Approach speeds of the athletes were not controlled by the researcher but instead were used as a guideline to ensure each trial was performed consistently for each athlete.

The knee joint angles for male and female soccer players are reported in Tables S3 and S4, respectively. A positive value denotes knee flexion, while a negative denotes knee extension (relative to the athlete's standing T-pose). For rotations around the longitudinal axis, a positive value denotes internal rotation, while a negative value represents external knee rotation. Knee varus is represented by a positive joint angle, while knee valgus is denoted by a negative value.

Hip rotation around the longitudinal axis, as well as hip abduction and adduction, are shown in Tables S5 and S6. A positive value denotes internal hip rotation about the longitudinal axis, as well as presenting hip abduction.

## 4. Discussion

The aim of the present study is to characterise the biomechanics of male and female soccer players, through motion capture analysis, over a variety of movements on an artificial grass playing surface. Studies have suggested that changes in joint angles during certain movements could lead to an increased risk of ACL injury in female soccer [12, 13]. Joint angles of interest, particularly in the “position of no return,” include internal rotation and adduction of the hip and an extended knee under valgus with an external tibial rotation [27]. This then formed the hypothesis that female players exhibited higher levels of knee valgus and hip adduction over a broad range of movements.

As shown in Table S3, female players landed with a high level of knee flexion at ground contact for a majority of movements, aside from the plant-and-cut movement, where the knee undergoes a level of extension. This extension removes the body's ability to effectively minimise the load through the lower limb as it would by progressively flexing the knee joint throughout the motion. This is notable as the plant-and-cut movement is one mechanism that the previous literature details as a high-risk movement for ACL injuries in females [27, 34]. Similarly, for a plant-and-cut movement, as well as a stop-turn movement, female players demonstrate large values of internal hip rotation, which Ireland [27] has highlighted as a potential factor for ACL injury.

In terms of knee internal/external rotation as well as varus/valgus rotation, female players tend to exhibit safer biomechanical angles for most movements aside from straight-line running. For running, female players exhibited both knee valgus and internal knee rotation which can place the lower limb in the “position of no return” and at a heightened risk of ACL injury. For both the sidestep and instep kick, as detailed in Table S4, females had larger knee valgus when compared to males, which can place added strain on the ACL. For the sidestep kick, females also experienced external knee rotation of similar magnitude to the measurements of Kellis et al. [35]. This external rotation can place direct strain on the ACL due to the femoral position relative to a stationary tibia.

At the ankle, female players performed actions with a dorsiflexed foot, indicating a tendency to land on the heel when striking the ground. One exception, however, was for a jump-exit movement whereby female players would land with their foot in a plantarflexed position before accelerating at a 45° angle. This plantarflexion could indicate a shift forward in body weight, which, when coupled with the level of hip abduction experienced in the same movement, could be a potential indicator for injury risk.

From the PCA, two key joint angles were found to have the largest impact on the variance between joint angles, namely the internal and external rotation of the knee joint about the longitudinal axis, as well as the abduction and adduction of the hip joint. The rotation of the knee joint is of particular importance as it relates directly to non-contact ACL injury. This is evident in the fact that the knee is one of the most injured joints among soccer players [7]. The influence of hip abduction and adduction angle could correlate with several anatomical and muscular differences between male and female athletes. This result, however, disproves the hypothesis that knee valgus is the underlying factor in female player biomechanics, supporting the research of Nilstad et al. [36] and Krosshaug et al. [29], who suggest that knee valgus may not play an influential role in the prediction of ACL injury risk.

While there has been an abundance of literature regarding the biomechanics of male soccer players, studies are often limited with regard to the number and nature of the performed movements as well as the settings in which they are performed. This significantly impacts the accuracy of such results as they do not accurately represent game-like scenarios.

Both males and females followed the same biomechanical behaviour at the hip joint throughout the different movements. As shown in Table S4, the male hip remained in a somewhat neutral position at ground contact aside from movements that included an angled acceleration, such as the run turn, plant cut, and jump exit movements. In these movements, at ground contact, the hip was rotated internally with abduction. For the run-turn and plant-cut movements, this was coupled with an internal knee rotation, highlighting a potential point of foot fixation as the hip rotates relative to the shank, potentially placing the athlete in the “position of no return” [27]. The magnitude of the hip rotation and abduction angles, particularly during the plant-cut movement, are comparable to those obtained by Dos'Santos et al. [37].

For the instep kick, male players exhibited a degree of knee valgus as well as internal rotation, both of which are factors that have links to ACL strain. While these values are similar in magnitude to the measurements of Kellis et al. [35], they are less than those obtained in a plant cut or a run stop motion, both of which have been identified as high-risk movements [13].

Male soccer players tended to exhibit some level of knee flexion across all the tested movements, a factor Ireland [27] identifies as beneficial for the reduction in risk of ACL injury. While males exhibited flexion across all movements, the magnitude was often less than their female counterparts, indicating a tendency to perform movements in more of a neutral position.

In terms of ankle positioning, male players performed movements with an approximately neutral ankle in the frontal plane with minimal inversion or eversion across all movements. For jumping movements, players exhibited plantarflexion, indicating an inclination to land on the toes rather than the heel. This could imply that the players' body-weight may be shifted forward, away from the frontal plane, which poses a potential risk for injury [27]. In contrast, for movements where the player had an incoming velocity, the foot was more dorsiflexed, indicating a heel strike and a weight shift behind the frontal plane.

For male players, the PCA showed that the internal and external rotation of the knee, coupled with the internal and external rotation of the hip, had the largest influence on the variance of the remaining joint angles. While the influence of the knee rotation was consistent between sexes, the change from hip abduction to hip rotation could be partly explained by the decrease in the activation of abductor muscles compared to female athletes, as demonstrated by Lewis et al. [38]. This highlights the need for coaches and strength training personnel to employ an individualised approach when considering the differences in movement between male and female players. These rotations about the knee and the hip occur along the longitudinal axis of the body. Movements about this axis have been explored with regard to the shoe–surface interaction of players to avoid fixation-related injuries. To help mitigate the increased risk of injury early in the season, as discussed by Orchard [39], coaches should avoid intense, rotation-based movements until female players are adequately prepared and warmed up. As the axis of rotation for the key movements at the hip and knee is the same as the shoe–surface interaction, this proposes a potential link between lower limb movement, foot fixation, and injury risk, thus highlighting the importance rotational traction plays in lower limb injury risk and how significant selecting the right boots for the surface conditions may be [40–42].

As evidenced by the standard deviation of results and the exclusion of eight movements from the PCA, the sample size of the research was a limiting factor. While variations within each of the four trials of the same player were relatively minor, the variation between players of the same sex was, for some manoeuvres, significant. Despite these limitations, the study methodology was supported by approach speeds and actions performed in the literature. While no direct instruction was given to participants with respect to their approach speed, the value was recorded to compare against the speed of movement reported in the literature. The straight-line running speeds, while not near the magnitude of the top speeds typical in professional sport, are reminiscent of the speeds athletes are performing at for 30% of the game, while high-speed running accounts for only 10% of game actions [43]. Plant and cut approach speeds in this research were consistent with other tests conducted by Besier et al. [44], Rovan et al. [45], and Suzuki et al. [46].

To help understand the lower limb movements of both male and female soccer players, a 3D motion capture analysis of players was performed on an artificial grass surface for a variety of movement types. Previous studies have shown that the surface type, as well as player sex, can affect lower limb biomechanics and, thus, the potential risk of ACL injuries for soccer players. It was found that while the specific joint angles were dependent on the action being performed, certain movements did follow similar trends in the data. For example, movements involving an acceleration phase (plant-cut, jump-exit, and run-turn) showed higher levels of hip movement for both male and female soccer players. Similarly, in jumping movements, male players landed on a dorsiflexed foot, while for running-based movements, they exhibited some degree of plantarflexion. These trends, when considered holistically at the ankle, knee, and hip, could provide insight into the potential risk of ACL injuries and possibly highlight the cause as to the disparity between male and female ACL injury rates.

## Figures and Tables

**Table 1:** The component matrix generated from the PCA for the male sidekick movement.

	Component	
	1	2
Knee internal/external rotation	0.883	0.264
Ankle inversion/eversion	0.922	−0.076
Ankle plantarflexion/dorsiflexion	0.777	−0.010
Knee flexion/extension	−0.694	0.534
Knee varus/valgus	−0.239	0.912
Hip abduction/adduction	−0.074	−0.798
Hip internal/external rotation	−0.539	−0.679

## Data Availability

The participants of this study did not give written consent for their data to be shared publicly, so due to the sensitive nature of the research, supporting data are not available.

## Supplementary Materials

Supplementary Materials The supplementary material includes biomechanical angles and values recorded at ground contact for various movements of male and female players. Tables S1–S6 detail the average of the biomechanical angles and values for particular actions at ground contact and are separated by sex and joint location. It also reports the PCA analysis for the movements that satisfied the criteria with respect to the K–M–O test of sphericity as well as the level of significance.
