# Supplementary material for: 3D Kinematics of Male and Female Soccer Players for a Variety of Game-Specific Skills
Source: Appl Bionics Biomech. 2024 Jan 8;2024:9588416. doi: 10.1155/2024/9588416 (PMC10789512; doi:10.1155/2024/9588416)
Supplement: Supplementary Materials — The supplementary material includes biomechanical angles and values recorded at ground contact for various movements of male and female players. Tables S1–S6 detail the average of the biomechanical angles and values for particular actions at ground contact and are separated by sex and joint location. It also reports the PCA analysis for the movements that satisfied the criteria with respect to the K–M–O test of sphericity as well as the level of significance. [file 9588416.f1.docx]

# Supplementary Material

Tables S1-S6 detail the average of the biomechanical angles and values for particular actions at ground contact and are separated by sex and joint location.

|  | Biomechanical Values | | |
| --- | --- | --- | --- |
| Action | *Approach Speed (m/s)* | *Inversion/Eversion (⁰)* | *Plantar/Dorsiflexion (⁰)* |
| *Running* | 3.81 (0.45) | -0.86 (1.29) | 0.55 (9.83) |
| *Run Stop* | 2.35 (0.43) | -2.68 (1.99) | -32.55 (3.58) |
| *Run Turn* | 2.19 (0.54) | 2.49 (5.29) | -10.16 (11.17) |
| *Plant Cut* | 3.34 (0.39) | -0.70 (3.63) | -9.25 (5.88) |
| *Jumping* | N/A | 1.84 (2.57) | 16.37 (2.71) |
| *Jump Exit* | N/A | -0.49 (4.10) | 18.73 (8.29) |
| *Sidestep Kick* | N/A | -0.14 (0.69) | -9.83 (9.00) |
| *Instep Kick* | N/A | -1.42 (3.35) | -11.46 (2.62) |

Table S1: The mean and standard deviation over the 4 trials of the approach speed and joint angles at the ankle of male soccer players performing a variety of actions. Positive values correspond to Inversion and Plantarflexion.

Table S2: The mean and standard deviation of the approach speed and joint angles at the ankle of female soccer players performing a variety of actions. Positive values correspond Inversion and Plantarflexion.

|  | Biomechanical Values | | |
| --- | --- | --- | --- |
| Action | *Approach Speed (m/s)* | *Inversion/Eversion (⁰)* | *Plantar/Dorsiflexion (⁰)* |
| *Running* | 3.91 (0.57) | -0.87 (2.10) | 0.27 (5.52) |
| *Run Stop* | 2.89 (0.44) | -3.65 (11.02) | -25.95 (4.51) |
| *Run Turn* | 1.64 (0.47) | 1.73 (5.54) | -0.85 (13.28) |
| *Plant Cut* | 3.54 (0.39) | 2.08 (6.26) | -11.92 (7.86) |
| *Jumping* | N/A | -2.38 (3.29) | -14.18 (7.20) |
| *Jump Exit* | N/A | -0.99 (4.38) | 20.75 (9.92) |
| *Sidestep Kick* | N/A | -0.51 2.07) | -0.70 (5.45) |
| *Instep Kick* | N/A | -1.92 (2.19) | -7.17 (5.68) |

Table S3: The mean and standard deviation of joint angles at the knee of male soccer players performing a variety of actions. Positive values correspond to knee flexion, internal knee rotation and knee varus.

|  | Biomechanical Values | | |
| --- | --- | --- | --- |
| Action | *Flexion/Extension (⁰)* | *Internal/External Rotation (⁰)* | *Varus/Valgus (⁰)* |
| *Running* | 8.79 (6.73) | -1.75 (10.61) | 4.43 (3.10) |
| *Run Stop* | 7.59 (5.72) | 9.93 (12.44) | 9.51 (5.81) |
| *Run Turn* | 14.75 (6.29) | 6.18 (8.35) | 13.20 (10.27) |
| *Plant Cut* | 14.01 (5.60) | 5.26 (15.60) | 3.73 (9.37) |
| *Jumping* | 22.65 (10.28) | -0.10 (7.57) | 7.93 (10.94) |
| *Jump Exit* | 16.49 (12.90) | 1.56 (6.34) | 0.69 (11.83) |
| *Sidestep Kick* | 5.44 (12.03) | 1.74 (6.12) | 9.24 (6.60) |
| *Instep Kick* | 13.88 (9.07) | 5.15 (6.34) | 6.87 (4.66) |

Table S4: The mean and standard deviation of joint angles at the knee of female soccer players performing a variety of actions. Positive values correspond to knee flexion, internal knee rotation and knee varus.

|  | Biomechanical Values | | |
| --- | --- | --- | --- |
| Action | *Flexion/Extension (⁰)* | *Internal/External Rotation (⁰)* | *Varus/Valgus (⁰)* |
| *Running* | 19.17 (7.10) | 5.82 (8.53) | -2.37 (7.12) |
| *Run Stop* | 13.46 (7.30) | -5.60 (11.92) | 0.89 (11.19) |
| *Run Turn* | 26.09 (11.19) | 0.09 (12.74) | 7.45 (10.00) |
| *Plant Cut* | -10.71 (9.16) | 0.55 (11.56) | 4.57 (6.14) |
| *Jumping* | 35.39 (19.74) | -2.48 (13.32) | 2.24 (16.34) |
| *Jump Exit* | 30.40 (15.77) | -4.90 (4.90) | 2.87 (12.25) |
| *Sidestep Kick* | 17.06 (11.63) | 0.88 (11.48) | 4.15 (8.00) |
| *Instep Kick* | 18.71 (9.30) | -3.35 (9.02) | 4.73 (10.20) |

Table S5: The mean and standard deviation of joint angles at the hip of male soccer players performing a variety of actions. Positive values correspond to internal hip rotation and hip abduction.

|  | Biomechanical Values | |
| --- | --- | --- |
| Action | *Internal/External Rotation (⁰)* | *Abduction/Adduction (⁰)* |
| *Running* | 1.27 (9.50) | -11.96 (4.78) |
| *Run Stop* | 9.75 (7.61) | 7.42 (10.40) |
| *Run Turn* | 11.34 (15.07) | 15.38 (8.54) |
| *Plant Cut* | 18.14 (12.52) | 0.24 (14.95) |
| *Jumping* | -1.25 (6.99) | 5.46 (8.29) |
| *Jump Exit* | 1.36 (9.67) | 12.14 (10.56) |
| *Sidestep Kick* | 1.19 (8.53) | -2.59 (7.44) |
| *Instep Kick* | 7.43 (8.16) | -9.43 (9.04) |

Table S6: The mean and standard deviation of joint angles at the hip of female soccer players performing a variety of actions. Positive values correspond internal hip rotation and hip abduction.

|  | Biomechanical Values | |
| --- | --- | --- |
| Action | *Internal/External Rotation (⁰)* | *Abduction/Adduction (⁰)* |
| *Running* | -0.54 (6.65) | -0.74 (7.47) |
| *Run Stop* | 2.44 (11.44) | 1.77 (7.30) |
| *Run Turn* | 19.24 (9.28) | -9.60 (12.98) |
| *Plant Cut* | 13.88 (9.54) | 10.02 (12.60) |
| *Jumping* | -5.31 (10.78) | 1.55 (12.62) |
| *Jump Exit* | -1.96 (7.73) | 11.34 (8.09) |
| *Sidestep Kick* | -2.53 (7.44) | 1.74 (5.68) |
| *Instep Kick* | -12.43 (5.86) | 4.75 (7.29) |

***The Complete PCA for each of the applicable movements***

As mentioned, movements (e.g. male straight line running followed by a sudden stop) were not included in the PCA as they had a KMO of less than 0.5. To improve legibility, components with values of less than 0.32 were excluded from these component tables as recommended by Tabachnick, Fidell ^47^. The correlation matrices of these movements, as well as their significance values have also been recorded.

***Jump Exit - Female***

***Jump Exit - Male***

***Jump - Male***


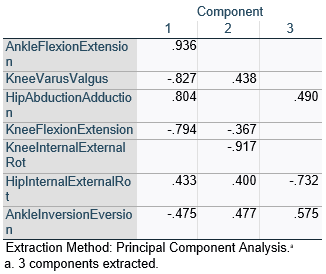

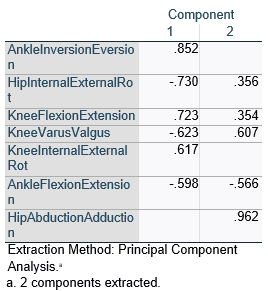

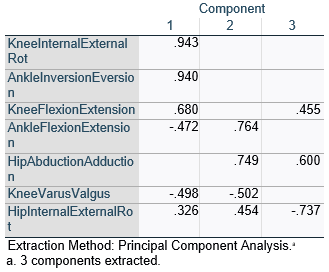


***Run and turn -Male***


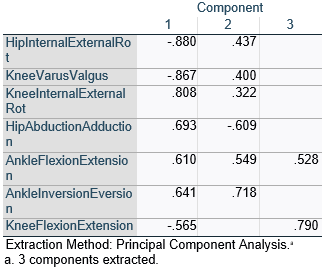

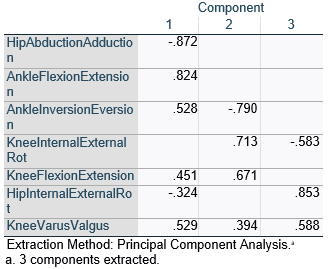


***Run - Female***


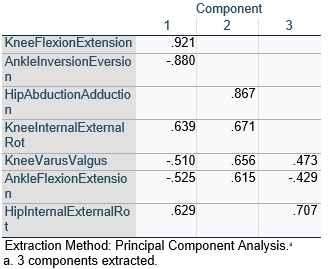


***Run - Male***

***Sidestep kick -Male***


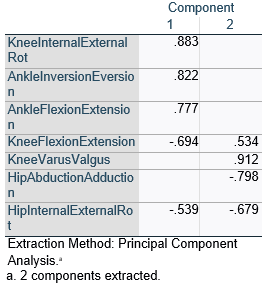


***Sidestep kick -Female***


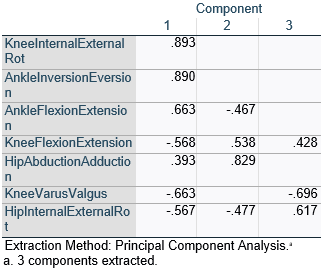


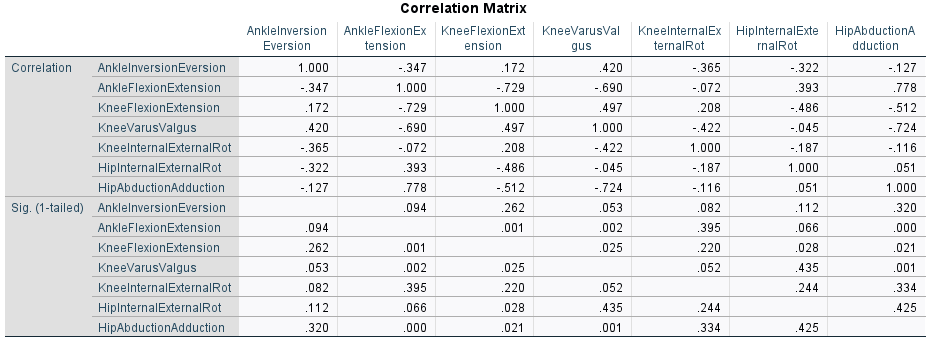


***Jump Exit - Female***


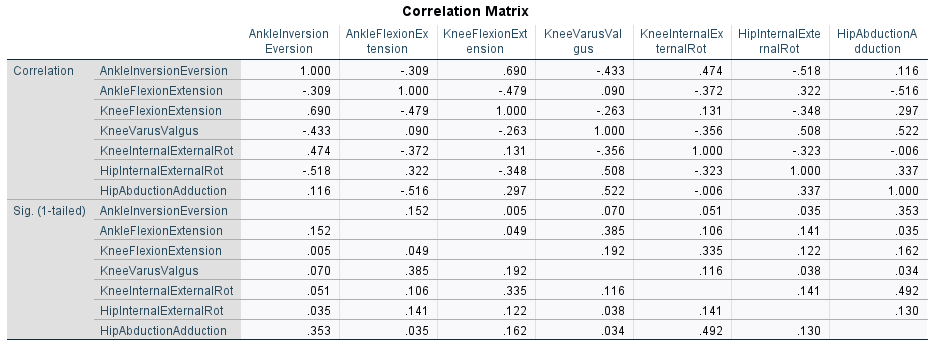


***Jump Exit - Male***


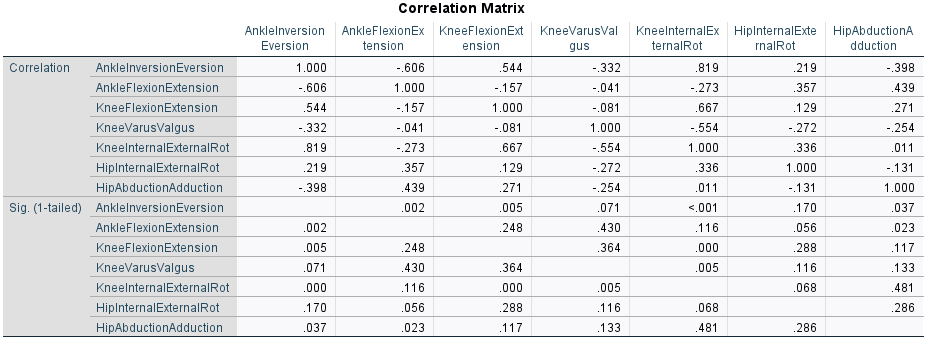


***Jump - Male***


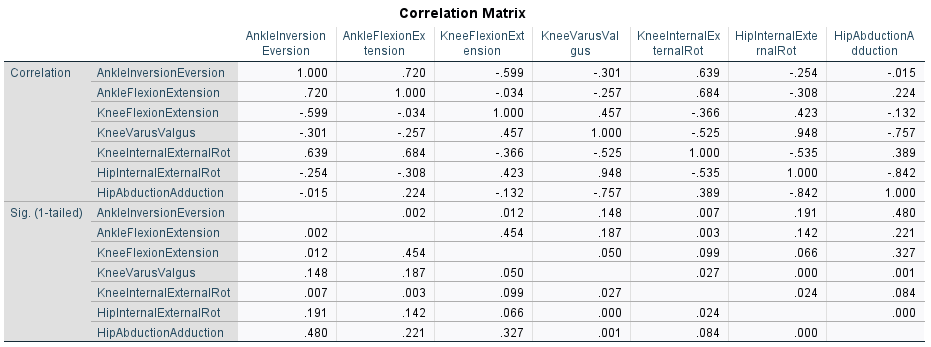


***Run and Turn - Male***


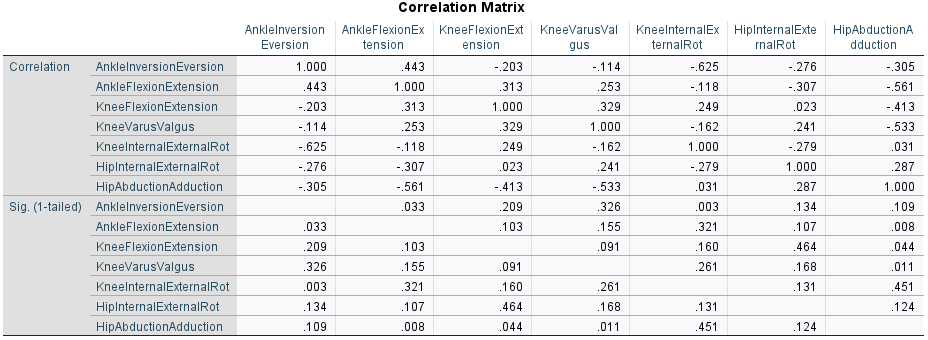


***Run - Female***

***Run - Male***


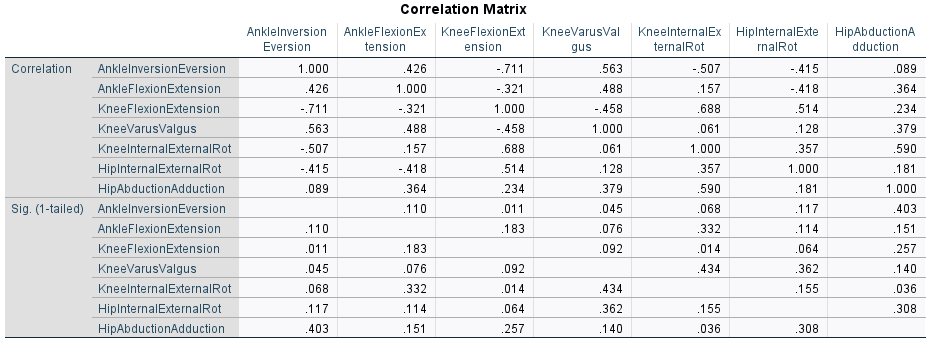

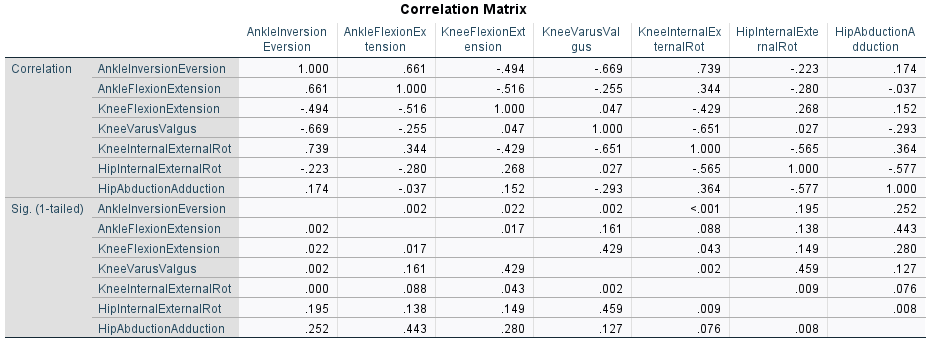


***Sidestep Kick - Female***


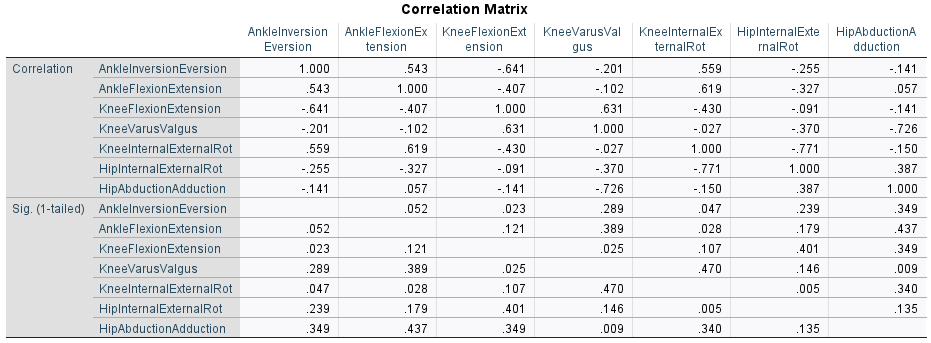


***Sidestep Kick - Male***
